# Investigation of the Rheological Properties and Chemical Structure of Asphalt under Multiple Aging Conditions of Heat, UV and Aqueous Solution

**DOI:** 10.3390/ma15165711

**Published:** 2022-08-19

**Authors:** Yingxue Zou, Ling Pang, Shi Xu, Shaopeng Wu, Miao Yuan, Serji Amirkhanian, Haiqin Xu, Yang Lv, Xiang Gao

**Affiliations:** 1 State Key Laboratory of Silicate Materials for Architectures, Wuhan University of Technology, Luoshi Road 122, Wuhan 430070, China; 2 School of Civil Engineering and Architecture, Wuhan University of Technology, Luoshi Road 122, Wuhan 430070, China; 3 Faculty of Civil Engineering and Geosciences, Delft University of Technology, Stevinweg 1, 2628 CN Delft, The Netherlands; 4 Foshan Transportation Science and Technology Co., Ltd., Foshan 528315, China; 5 Department of Civil Construction and Environmental Engineering, University of Alabama, Tuscaloosa, AL 35487, USA

**Keywords:** asphalt, thermal-oxygen aging, UV aging, moisture damage, rheological performance, chemical structure

## Abstract

During the service period, asphalt materials are affected by various natural factors, including heat, ultraviolet light, oxygen and moisture, etc., resulting in the reduction of pavement performance, the increase of pavement distress and shortening of service life. This study aims to investigate the aging performance of asphalt under multiple aging conditions of heat, UV and aqueous solution. Thermal-oxygen aging, UV aging and hydrostatic erosion tests were carried out sequentially on asphalt. The rheological properties, chemical structure and element composition of asphalt were characterized before and after aging, and the effect mechanism of multiple conditions was discussed. The results show that the multiple conditions of heat and UV can increase the rutting resistance and weaken the cracking resistance of asphalt. However, the effect degree of UV decreases gradually with the deepening of aging degree. Additionally, the effect of water on the physicochemical properties is less than that of UV; however, water can increase the sensitivity of physicochemical properties to UV. In summary, this study explored the short-term cycling effect of heat, light and water on asphalt and provided an idea for simulation test of asphalt under multiple aging condition.

## 1. Introduction

Asphalt concrete, due to its advantages of easy construction, driving comfort and low noise, is widely used as a paving material throughout the world [1,2]. The appearance of asphalt concrete distress is mainly due to the occurrence of aging under the conditions of heat, oxygen, ultraviolet and water [3]. Thermal-oxygen aging occurs mainly during the construction period, including mixing, paving, etc. Its mechanism mainly includes the volatilization and oxidation of light components, the condensation of saturates and the degradation of macromolecules in asphalt [4]. UV radiation has the most significant effect on asphalt concrete aging during service. The molecular chains in asphalt absorb enough energy from the UV wavelengths to cause the bonds to break [5]. Additionally, water and the oxygen in the water can cause the alternating occurrence of asphalt oxidation, dissolution and migration [6]. In different regions with different climatic conditions, asphalt concretes are subjected to the diverse service environment [7]. Asphalt concrete in different areas are exposed to various corrosive media, such as salt, acid and alkali [8,9,10,11]. Asphalt aging is the main cause of asphalt concrete aging [12]. Therefore, there are a large number of studies are concerned with the aging of asphalt, but preliminary research mainly focused on single factor of asphalt aging due to the complexity of asphalt composition [13,14,15].

With the deepening of research, multiple factors are gradually being considered in the simulation of asphalt aging. Abouelsaad and White pointed out that the performance of hot mix asphalt mixture gradually weakened with the increase of coupling aging time [16]. Tan and Li investigated the coupled effect of thermal-oxygen–UV on asphalt and found the effect of coupled aging was more obvious than thermal-oxygen aging, which showed a rapid decay of asphalt properties [17]. Li et al. supposed that the coupled ageing mechanism of UV and different aqueous solution mainly includes three channels, including the excitation and break of asphalt molecules and the dissolution and separation of the organic component in asphalt [18]. It was Ilaria and Eyad who found that the combined effect of UV–heat–oxygen–moisture caused a portion of the asphalt to become soluble and dissolved, while the rest of the asphalt showed cracking [19]. Zhang et al. concluded that coupled aging effect of heat–UV–water was considered to have a significant effect on the viscoelasticity and high-temperature performance of warm mix asphalt [20]. These atmospheric factors had a very important role in the degradation and microstructural evolution of asphalt. However, the above studies simulate the simultaneous action of multiple factors on asphalt, and the interaction and contribution of each factor is not clear. Because the aging mechanism of the three kinds of aging methods is various, so is the effect behavior on properties of asphalt. The study of asphalt aging mechanism is important not only for predicting the longevity of asphalt, but information about asphalt aging can help correctly restore asphalt properties with recycled asphalt pavement (RAP). The industries all over the world are looking for solutions to use 100% RAP, which can significantly improve economic and ecological outcomes [21]. Therefore, it is of great interest to investigate the aging performance of asphalt under multiple conditions of heat, UV and aqueous solution. 

**Figure 1:**
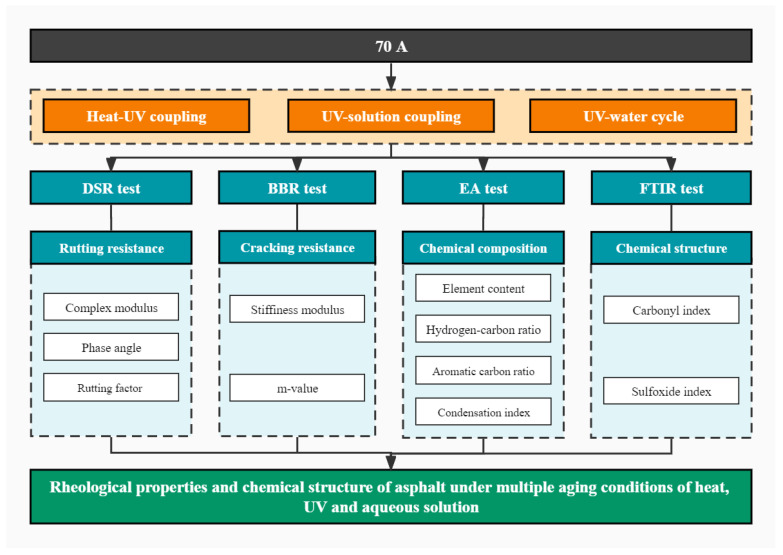
Research program.

Changes in asphalt properties are mainly caused by changes in the internal chemical composition and structure. The combination of EA and FTIR can complement each other and facilitate a more accurate analysis of the microstructure of asphalt after aging [22]. Therefore, the aging performance of asphalt under multiple conditions of heat, UV and aqueous solution was investigated in this study using the characterization of the rheological properties, element composition and chemical structure of asphalt, and the performance evolution law was discussed. The research program is illustrated in Figure 1. This study adopted sequentially Thin Film Oven Test (TFOT), UV aging test and hydrostatic immersion test to conduct different aging test on 70 A. The multiple aging conditions are divided into three levels, including heat–UV coupling, UV–solution coupling and UV–water cycle. After aging, Dynamic Shear Rheometer (DSR) test and Bending Beam Rheometer (BBR) test were employed to observe the change of rheological properties, including high-temperature rutting resistance and low-temperature cracking resistance. Additionally, the major element composition and the characteristic functional groups of asphalt were detected by EA test and FTIR test, respectively. The results of EA test and FTIR test were combined to analyze the changes in chemical composition before and after aging and to investigate the aging mechanism of the properties changes.

## 2. Materials and Experiments

### 2.1. Materials

#### 2.1.1. Asphalt

Base asphalt with 60/80 penetration grade (simply referred as 70 A) employed in this study was obtained from Hubei Guochuang Road Material Technology Co., Ltd. (Wuhan, China). The basic physical properties were illustrated in Table 1.

#### 2.1.2. Preparation of Aqueous Solution

Four kinds of aqueous solutions with different media were prepared, including distilled water, 10 wt% NaCl saline solution, pH 3 acid solution and pH 11 alkali solution, to simulate asphalt immersed in various aqueous solution. The 10 wt% NaCl saline solution was obtained by dissolving solid sodium chloride with distilled water. The distilled water was used to dilute the mixed solution of sulfuric acid and nitric acid with a molar ratio of 9:1, and the pH 11 alkali solution was prepared by dissolving a certain amount of solid sodium hydroxide. The pH value of which was monitored by a precision pH meter.

### 2.2. Aging Simulation Test of Asphalt

**Figure 2:**
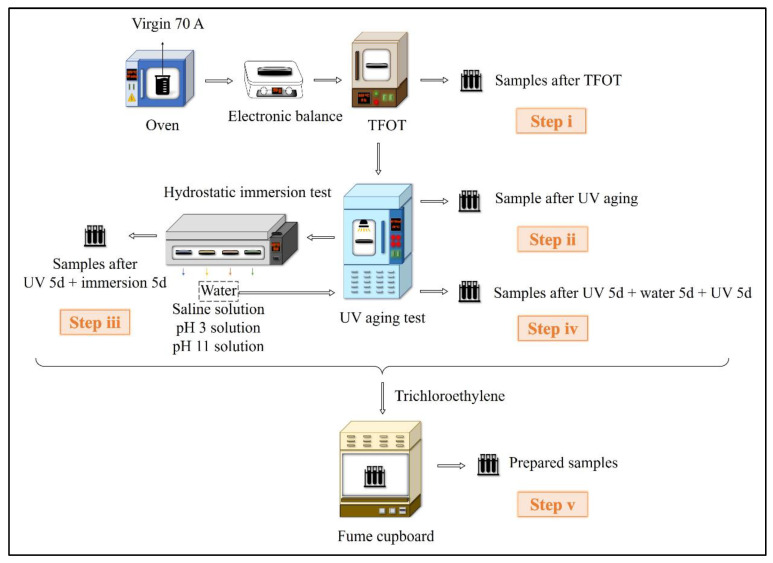
Sample preparation procedure.

Figure 2 illustrates the sample preparation procedure under the multiple conditions of heat, UV and aqueous solution following six steps:

- The 50 g of 70 A was poured on a dry aging tray with a diameter of 140 mm, then the aging tray was put into a thermal film oven at 163 °C for 5 h to obtain TFOT aged samples;
- The TFOT aged samples were placed in UV aging oven at 50 W/m^2^ of irradiation intensity and 60 °C for 5 days, 10 days and 15 days to obtain UV aged samples;
- The UV-aged samples were treated by hydrostatic immersion experiments in a water bath at 60 °C for 5 days with distilled water, 10 wt% NaCl salt solution, pH 3 acid solution and pH11 alkali solution to obtain the samples (UV 5d + immersion 5d);
- The sample (UV 5d + water 5d) were subjected to UV aging for another 5 days, according to step iii, to obtain the samples (UV 5d + water 5d + UV 5d);
- Trichloroethylene was selected as a solvent to dissolve the upper part of the samples at each stage for 90 s, and the trichloroethylene-asphalt solution was poured into a clean container then placed in fume cupboard for 72 h to allow the trichloroethylene to evaporate completely. Finally, the residues were collected as the aged asphalt samples.

### 2.3. Characterization of Asphalt

The rheological properties, chemical structure and element composition of asphalt were characterized by DSR, BBR, EA and FTIR, and the related information of the instruments as shown in the Table 2. These tests were performed in accordance with relevant specifications.

#### 2.3.1. DSR Test

As a kind of viscoelastic material, asphalt is sensitive to changes in temperature and load. The rheological property of asphalt has a vital effect on its processability and pavement performance of asphalt mixture [31]. The high-temperature rheological property of asphalt samples was characterized by a temperature sweep using DSR with strain control mode. About 0.8 g of asphalt sample was used to prepare the cylinder with a diameter of 25 mm and a height of 1 mm. The complex modulus (G*) and phase angle (δ) of asphalt are the main parameters. The high-temperature rheological property is usually characterized by the rutting factor (G*/sinδ) to evaluate the rutting resistance [32]. The ratio of G*/sinδ before and after aging is defined as the Rutting factor Aging Index (RAI), which can quantify the influence of aging conditions on the high-temperature performance of asphalt, as shown in Equation (1) [33].

(1) $$\mathrm{RAI}=\frac{G_{\mathrm{aging}}^{*}/{\sin\delta }_{\mathrm{aging}}}{G_{\mathrm{virgin}}^{*}/{\sin\delta }_{\mathrm{virgin}}}$$

where G*_virgin_ and G*_aging_ represent the G* of asphalt before and after aging, respectively, Pa; and δ_virgin_ and δ_aging_ represent the δ of asphalt before and after aging, respectively. The greater the RAI, the more distinct the aging effect.

#### 2.3.2. BBR Test

As the evaluation parameters of cracking resistance, the creep Stiffness modulus (S) and creep rate (m-value) of asphalt were obtained by BBR test at low temperature, as shown in Equations (2) and (3) [34]. The sample size was 127 mm × 12.7 mm × 6.35 mm. The higher the S, the worse the low temperature ductility. The m-value represents the change rate of S, and the greater the value, the higher the relaxation rate and the more excellent the low temperature performance.

(2) $$S\left(t\right)=\frac{PL^{3}}{4bh^{3}∆\left(t\right)}$$

(3) $$m\left(t\right)=B+2C{\left[\mathrm{lg}\left(t\right)\right]}^{2}$$

where *S*(*t*) represents the creep stiffness at 60 s, MPa; *P* represents the test load, mN; *L*, *b* and *h* represent the span length, width and depth of samples, mm; Δ(*t*) represents the deflection of samples at 60 s; and *B* and *C* represent the regression coefficients of lg[*S*(*t*)] and lg[*m*(*t*)].

#### 2.3.3. EA Test

The variation of the element composition of asphalt was detected by elemental analyzer under multiple condition. The analysis principle is dynamic adsorption–desorption TCD measurement program [35]. The C, H, N, S and O elements are the main elements of asphalt, and the test mode of C, H, N and S elements was selected. The asphalt with a weight of 5 mg is burned under pure oxygen conditions, and then the gas produced by the combustion is measured. After homogenization, the gas is separated in chromatography within the separation zone and finally measured in the detection zone. Since the content of hetero atoms in asphalt is too low to be ignored, the content of O is obtained by subtracting the content of C, H, N and S elements from 100%.

The density method was employed to analyze the structural composition of asphalt. Three important indexes, the molar ratio of hydrogen–carbon (n(H)/n(C)), aromatic carbon ratio (*f_A_*) and condensation index (*C_I_*), were selected. The larger the n(H)/n(C), the more saturated hydrocarbons in the asphalt and the less the aging degree and vice versa, as shown in Equation (4) [36].

(4) n(H)/n(C)=11.92×[ω(H)/ω(C)]

where ω(H) and ω(C) represent the mass fraction of hydrogen and carbon atoms in all elements of asphalt, respectively, %. 

The *f_A_* reflects the ratio of aromatic carbon atoms to total carbon atoms. The larger the *f_A_*, the more ring structure, especially the aromatic rings, which means that there are more macromolecules and a greater degree of aging, as shown in Equations (5)–(8).

(5) ρ=1.4673−0.0431ω(H)

(6) $$M_{c/\rho }=1201/[\rho \times \omega (C)]$$

(7) $${\left(M_{c/\rho }\right)}_{c}=M_{c/\rho }-6[100-\omega (C)-\omega (H)]/\omega (C)$$

(8) $$f_{A}=0.09{\left(M_{c/\rho }\right)}_{c}-1.15n\left(H\right)/n(C)+0.77$$

where *ρ* represents the density of asphalt, g/cm^3^, and $M_{c/\rho }$ and ${\left(M_{c/\rho }\right)}_{c}$ represent the molar volume of individual carbon atom before and after correction, respectively, L/mol.

The *C_I_* represents the molecule condensation degree, and the larger the *C_I_*, the greater the molecule condensation degree and the more complex the ring structure, as shown in Equation (9).

(9) $$C_{I}=2-n\left(H\right)/n(C)-f_{A}$$

#### 2.3.4. FTIR Test

Under the multiple aging condition, the chemical structure of asphalt was detected by FTIR with OMNIC 6.2 software (Thermo Fisher). Firstly, asphalt with a weight of 0.1 g was dissolved by CS_2_ to prepare 5 wt% asphalt–CS_2_ solution. Then, two drops were placed on the KBr chip with a glue dropper, and the CS_2_ was completely vaporized by infrared light. Finally, the prepared samples were undertaken for the FTIR test. The carbonyl group and sulfoxide group are the main products of asphalt oxidation, and their indexes ($I_{C=O}$ and $I_{S=O}$) are usually used to quantify the aging degree of asphalt, which can be calculated using Equations (10) and (11) [37].

(10) $$I_{C=O}=A_{1700{\mathrm{cm}\;}^{-1}}/\Sigma A_{2000-600{\mathrm{cm}\;}^{-1}}$$

(11) $$I_{S=O}=A_{1030{\mathrm{cm}\;}^{-1}}/\Sigma A_{2000-600{\mathrm{cm}\;}^{-1}}$$

where $A_{1700{\mathrm{cm}\;}^{-1}}$ and $A_{1030{\mathrm{cm}\;}^{-1}}$ represent the area of carbonyl group and sulfoxide group at the 1700 cm^−1^ and 1030 cm^−1^, respectively, and $\Sigma A_{2000-600{\mathrm{cm}\;}^{-1}}$ represents the total area from 2000 cm^−1^ to 600 cm^−1^. The higher the indexes, the greater the degree of aging.

## 3. Results and Discussion

### 3.1. High-Temperature Rutting Resistance

**Figure 3:**
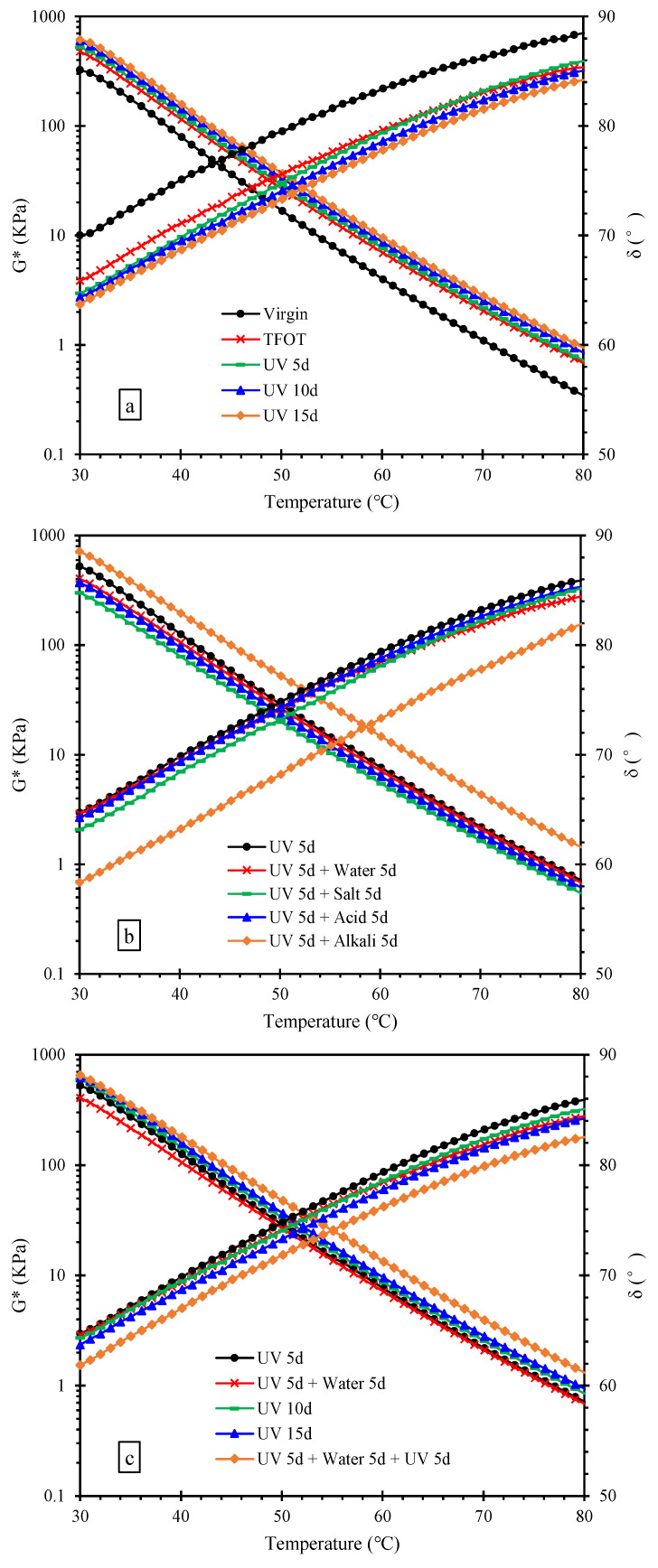
G* and δ of asphalt after the multiple aging of heat, UV and solution. (**a**) multiple aging of heat and UV; (**b**) multiple aging of heat, UV and solution; (**c**) aging cycle of UV and water.

The G* and δ of asphalt are the main parameters of the high-temperature rheological property of asphalt. The greater the G*, the greater the shear deformation resistance and vice versa. The δ can evaluate the ratio of elastic components and viscous components. The greater the δ, the greater the viscous components. Conversely, the smaller the δ, the greater the elastic components. Figure 3 shows that the G* and δ of asphalt after multiple aging through heat, UV and aqueous solution. As can be seen from Figure 3a, the G* and δ of asphalt aged with TFOT increased and decreased, respectively, indicating the increase in shear deformation resistance and elastic components. The trend was further deepened after UV aging, manifesting the effect of UV was accumulated on the sample after thermal-oxygen aging. As can be seen from Figure 3b, the G* of asphalt samples declined after immersion in water, saline solution and acid solution, while the G* of asphalt samples increased after immersion in alkali solution. The δ of asphalt decreased after immersion in the four kinds of solutions. This could be explained by that there were some oxidation products on the surface of samples (UV 5d), and the existence of moisture resulted in dissolution and migration of these, leading to the reduction of the shear deformation resistance during immersion in three kinds of solution except alkali solution [38]. However, the reduction of other components might lead to an increase in the relative content of crystalline wax, resulting in an increment in the elastic components [39]. Chloride ions in saline solution could promote the emulsification of asphalt, and the esterification reaction between acid and olefin in asphalt generated long chain isomerized alkanes. The chemical reactions increased the content of saturates, resulting in the further reduction of the shear deformation resistance. Among these, the effect of salt solution on the high-temperature rheological property of asphalt was greater than that of water and acid solution, which might be due to the crystallization of salt in asphalt [40]. Moreover, the saponification of alkali solution with asphalt accelerated the asphalt oxidation to generate more asphaltenes, resulting in an increasing of the shear deformation resistance and elastic components [41]. After aging cycle of UV and water, the G* and δ of asphalt are illustrated in Figure 3c. The order of G* from small to large was the following order, (UV 5d + water 5d) < (UV 5d) < (UV 10d) < (UV 15d) < (UV 5d + water 5d + UV 5d). It suggested that the effect of water and UV on shear deformation resistance was opposite due to asphalt dissolution and migration, but water can increase the sensitivity of the shear deformation resistance to UV. The order of δ from small to large is the following: (UV 5d + water 5d + UV 5d) < (UV 15d) < (UV 10d) < (UV 5d + water 5d) < (UV 5d); manifesting water can also increase sensitivity of the viscoelasticity to UV.

**Figure 4:**
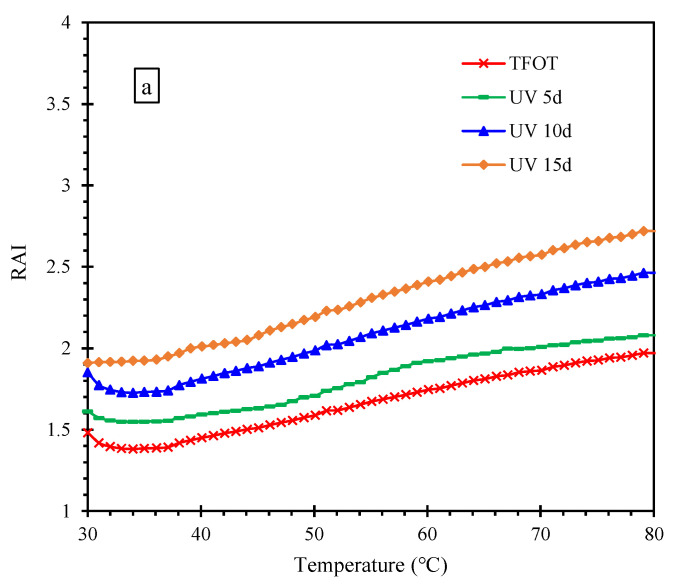

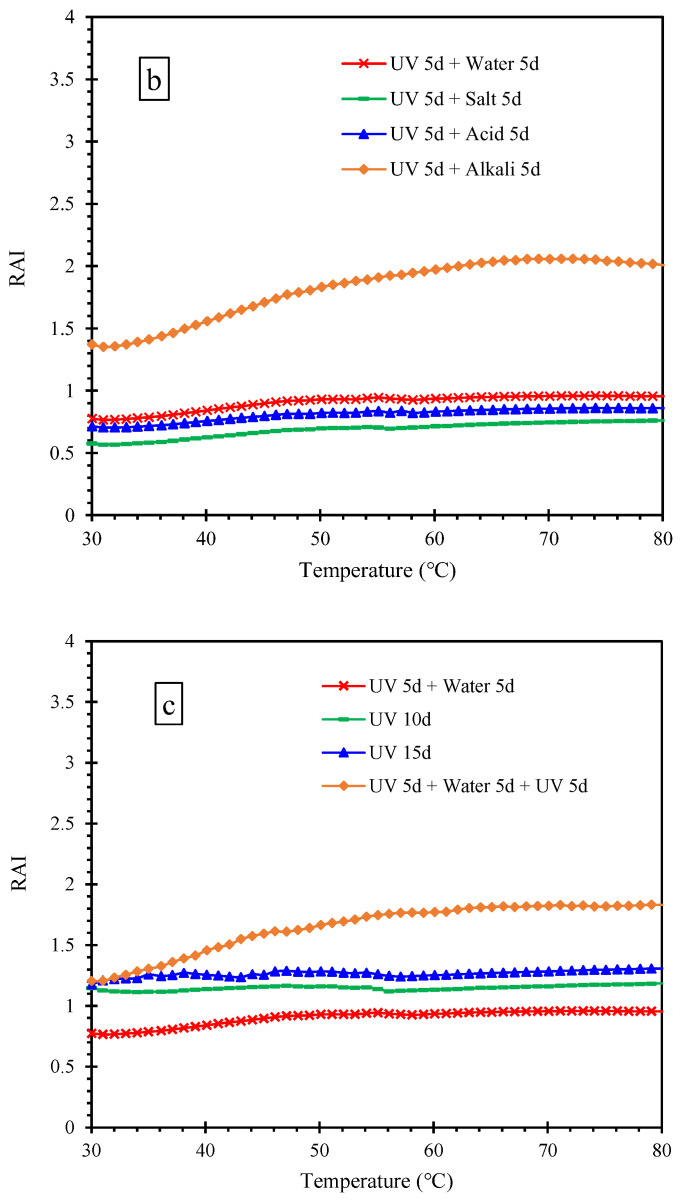
RAI of asphalt after the multiple aging of heat, UV and solution. (**a**) multiple aging of heat and UV; (**b**) multiple aging of heat, UV and solution; (**c**) aging cycle of UV and water.

Figure 4 shows the RAI of asphalt after the multiple aging. From Figure 4a, it can be seen that the RAIs of TFOT and UV aging were greater than 1 and increased with the increase of UV time. It indicates that the thermo-oxidative aging and UV aging have a positive effect on the rutting resistance of asphalt, and the positive effect could be accumulated. The RAI increased over temperature, suggesting that effect degree of thermal-oxygen and UV on the rutting resistance increased with the increment of temperature. Figure 4b,c illustrates the data graphs with the G*/sinδ of sample (UV 5d) as the $G_{\mathrm{virgin}}^{*}{/\sin\delta }_{\mathrm{virgin}}$. From Figure 4b, it can be seen that the RAIs of asphalt samples exposed to water, saline solution and acid solution were less than 1, while that of sample immersed in alkali solution was greater than 1. This demonstrates that the first three solutions had a negative impact on the rutting resistance of asphalt, while alkali solution improved the rutting resistance of asphalt. The RAIs of samples suffered the aqueous solutions except the alkali solution and were not affected by temperature, indicating they had similar effects on the rutting resistance at different temperatures. Additionally, the RAI of the asphalt treated by alkali solution increased with temperature, manifesting it had greater effects on the rutting resistance at high temperatures. From Figure 4c, it can be seen that the RAI of samples (UV 5d + water 5d) was less than that of samples (UV 10d), while the RAI of samples (UV 5d + water 5d + UV 5d) was greater than that of samples (UV 15d). It indicates that the effect of water on asphalt rutting resistance was less than UV, but it could increase the sensitivity of rutting resistance to UV. The RAI of samples (UV 5d + water 5d), (UV 10d) and (UV 15d) were not affected by temperature, manifesting water and UV had similar effects on the rutting resistance at different temperatures after UV aging for 5d. The RAI of samples (UV 5d + water 5d + UV 5d) increased with temperature, demonstrating that water made the rutting resistance more sensitive to temperature.

### 3.2. Low-Temperature Cracking Resistance

**Figure 5:**
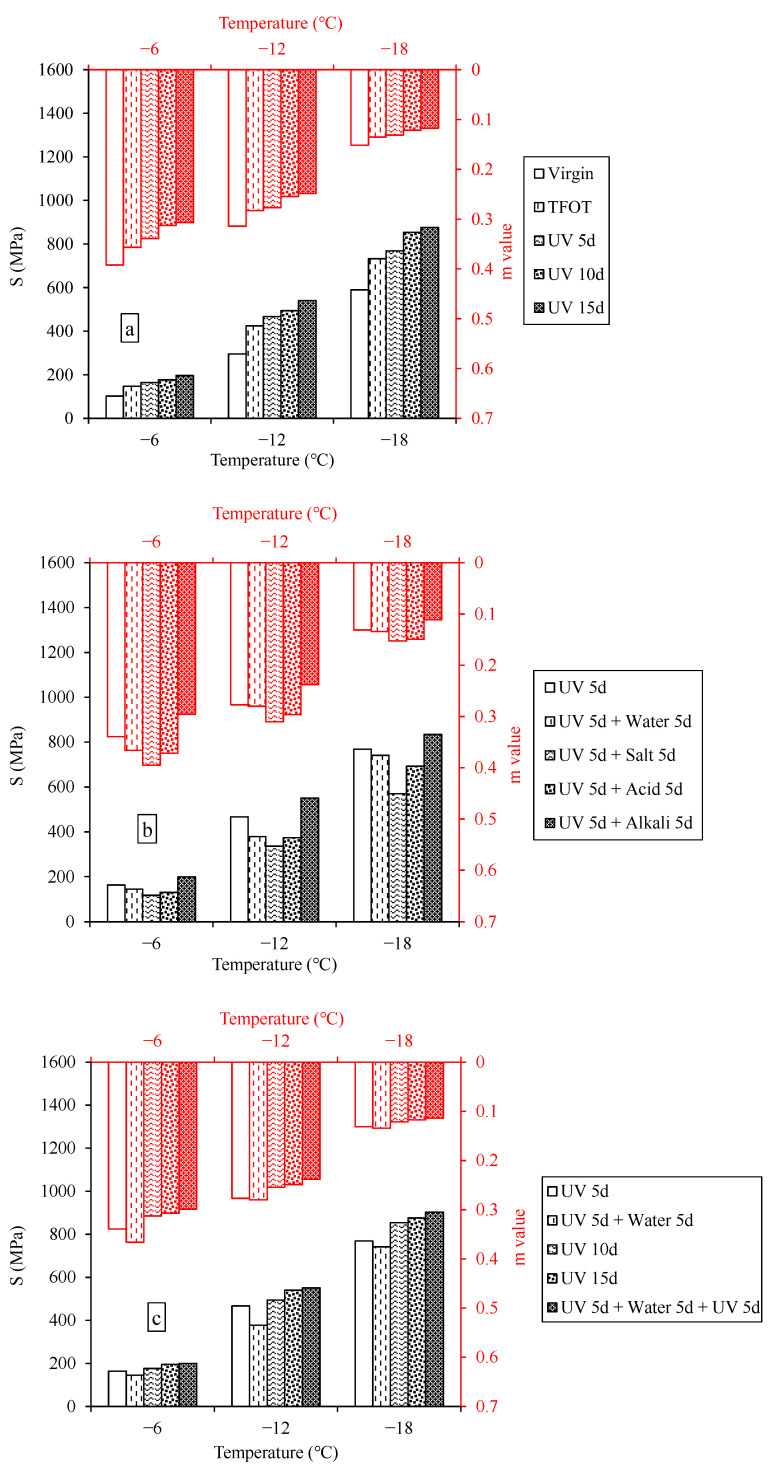
S and m-value of asphalt after the multiple aging of heat, UV and solution. (**a**) multiple aging of heat and UV; (**b**) multiple aging of heat, UV and solution; (**c**) aging cycle of UV and water.

Figure 5 shows that the S- and m-value of asphalt after the multiple aging of heat, UV and water. It can be seen that the S of asphalt increased and the m-value gradually decreased with the decrease of test temperature. It indicated that the low-temperature ductility and relaxation rate of the asphalt gradually decreased with the decreasing of temperature and the asphalt started to become hard and brittle. After thermal-oxygen aging, the S of samples gradually increase, while the m-value declined, manifesting the low-temperature, cracking resistance was weakened. The addition of UV aging deepened the change trend, meaning that thermal-oxygen and UV could accumulatively weaken the low-temperature cracking resistance. After the multiple aging of UV and aqueous solution, the S of asphalt exposed to water, saline solution and acid solution decreased and the m-value increased, while the opposite pattern appeared in samples suffered from alkali solution. This might be because the dissolution and migration of polar components in asphalt led to the softening of asphalt and enhancement of flexibility, leading to better cracking resistance at low temperatures. However, the polar components increased during immersion in alkali solution, hardening the asphalt samples and weakening the crack resistance at low temperature [42]. The rangeability order of S from small to large was the following: (UV 5d + water 5d) < (UV 5d) < (UV 10d) < (UV 15d) < (UV 5d + water 5d + UV 5d); and that of m-value was the opposite. It suggested that the effect of water was positive on cracking resistance, but water could increase the sensitivity of the cracking resistance to UV. 

### 3.3. Element Composition

**Figure 6:**
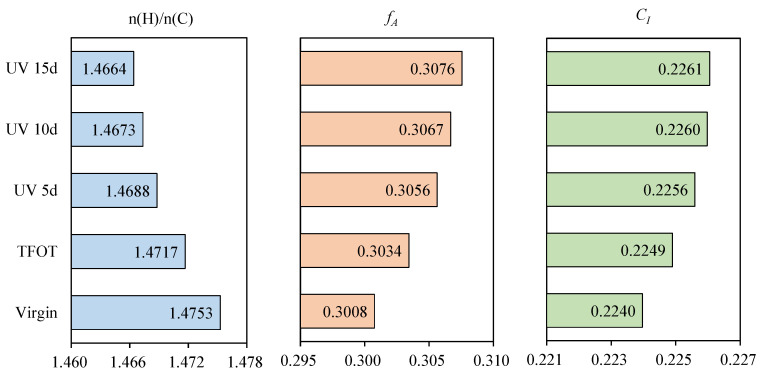
Important indexes (n(H)/n(C), *f_A_* and *C_I_*) of asphalt after the multiple aging of heat and UV.

Asphalt is a complex compound with a variety of polymeric hydrocarbons and their non-metallic derivatives, whose main constituent elements are carbon, hydrogen, oxygen, sulfur, nitrogen, etc. The effect of heat and UV on the element composition of asphalt samples were investigated to discuss the change mechanism in properties, as illustrated in Table 3. For 70 A, the elemental content of C and H was higher than that of N, S and O. After TFOT, the content of O increased, while that of others reduced, manifesting that oxidation reaction occurred to introduce oxygen atoms in air into the asphalt, therefore the relative content of other elements decreased. Moreover, the change trend of element composition increased gradually with the extension of UV aging time, indicating that the influence of UV accumulates gradually over time. The n(H)/n(C), *f_A_* and *C_I_* of asphalt after the multiple aging are displayed in Figure 6. The n(H)/n(C) declined, while the *f_A_* and *C_I_* increased after TFOT. The results showed that heat could decrease saturated hydrocarbon and increase the aromatic ring substance and its condensation degree, causing the existence of the more complex ring structure. The polymer molecule with the kind of aromatic ring as the main chain could not be rotated internally, resulting in the increasing of rigidity and the weakening of flexibility. As a result, the high-temperature rutting resistance was improved and the low temperature cracking resistance was weakened, as reflected in the DSR and BBR results. The trend was further driven by UV aging. Table 4 illustrated the comparison of rangeability in *C_I_* after different aging methods. It could be seen that the rangeability caused by TFOT was greater than that caused by UV 5d, and the rangeability gradually decreased with the extension of UV aging time, indicating that the sensitivity of elemental composition to UV decreased with the deepening of aging. 

**Figure 7:**
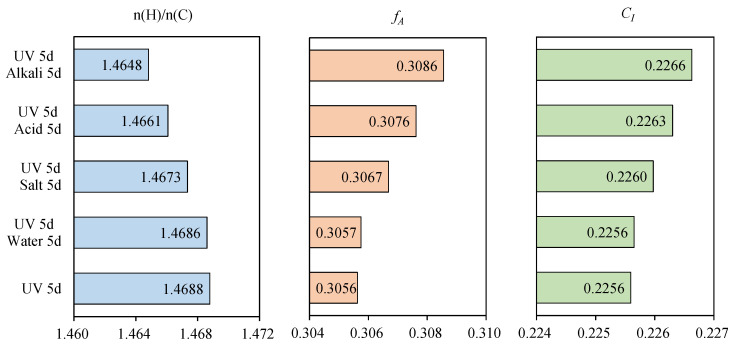
Important indexes (n(H)/n(C), *f_A_* and *C_I_*) of asphalt after the multiple aging of UV and aqueous solution.

Table 5 illustrated the element composition of asphalt samples after the multiple aging of UV and aqueous solution. Compared with UV 5d, the C and H remain essentially unchanged and the N and S declined to a certain extent, while the content of O increased after immersion in different aqueous solution. The n(H)/n(C) declined, while the *f_A_* and *C_I_* increased after immersion in different aqueous solution, as shown in Figure 7. It indicated that the oxidation during immersion caused the existence of molecules with high condensation degree, in which the water-soluble heterocyclic compounds containing N and S (including anhydrides, lactones and cyclic lactams) were easier to dissolve and migrate [42]. This resulted in the situation where N and S decreased but C and H remain essentially unchanged. The presence of solute could accelerate the trend, and the order of the effect degree is as follows: alkali > acid > salt > water.

**Figure 8:**
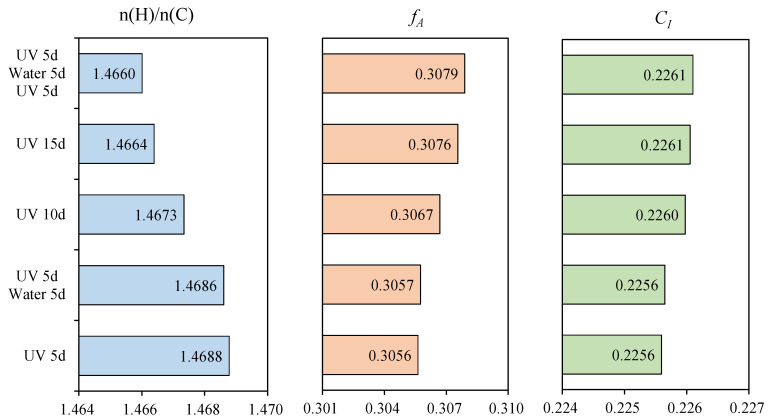
Important indexes (n(H)/n(C), *f_A_* and *C_I_*) of asphalt after the aging cycle of UV and water.

The element composition of asphalt was observed after the aging cycle of UV and water, as shown in Table 6. Compared to the sample (UV 10d), the sample (UV 5d + water 5d) had the same content of C, higher content of H and O and the lower content of N and S. The sample (UV 5d + water 5d + UV 5d) had the lower content of C and H, the same content of N and the higher content of S and O than the sample (UV 15d). Figure 8 describes the n(H)/n(C), *f_A_* and *C_I_* of asphalt after the aging cycle. The sample (UV 5d +water 5d) had the smaller n(H)/n(C), the greater *f_A_* and *C_I_* than the sample (UV 10d), meaning that the effect of water on element composition was less than UV. Compared to the sample (UV 15d), the n(H)/n(C) of sample (UV 5d +water 5d + UV 5d) was smaller but the *f_A_* and *C_I_* were greater. In addition, it could be seen from Table 4 that the rangeability of *C_I_* between the sample (UV 5d + water 5d and UV 5d) and the sample (UV 5d + water 5d) was 1.25 times that between the sample (UV 10d) and the sample (UV 5d), demonstrating that water could increase the sensitivity of element composition to UV. 

### 3.4. Chemical Structure

**Figure 9:**
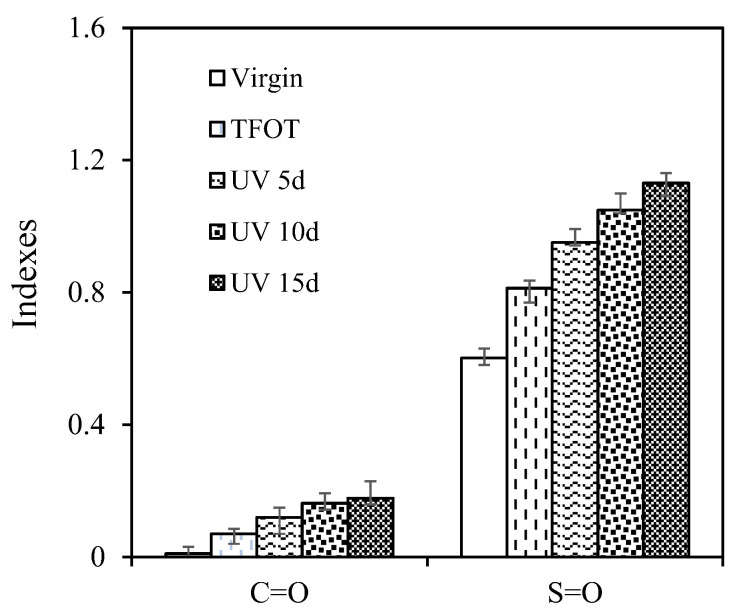
*I_C=O_* and *I_S=O_* of asphalt after the multiple aging of heat and UV.

The change of element composition and chemical structure are the fundamental reason for the change of rheological properties. The effect of multiple aging on chemical structure were characterized by FTIR test. Figure 9 illustrates that the *I_C=O_* and *I_S=O_* of asphalt after the multiple aging of heat and UV. The *I_C=O_* of virgin asphalt was close to 0, while the *I_S=O_* was far greater than the *I_C=O_*. It shows that the virgin asphalt was not oxidized and some parts of S element belong to S=O of asphalt itself [43]. Compared with the virgin, the *I_C=O_* of samples increased by 0.06, 0.11, 0.15 and 0.17, while the *I_S=O_* samples increased by 0.21, 0.35, 0.45 and 0.53 after TFOT aging, UV 5d, UV 10d and UV 15d. The coupling of thermal-oxygen and UV could accumulate to promote the asphalt oxidation. It indicated that asphalt aging increased polar functional groups, such as C=O and S=O, which had permanent dipoles and generate electrostatic force, resulting in the increase in the intermolecular friction resistance of asphalt [44]. The high-temperature rutting resistance was improved, and the low-temperature cracking resistance was weakened, which was shown in the DSR and BBR results. The rangeability of *I_C=O_* and *I_S=O_* under different aging methods was compared in Table 7. It could be seen that the rangeability caused by TFOT was greater than that caused by UV 5d, and the rangeability gradually decreased with UV aging time, indicating the sensitivity of the asphalt oxidation to UV decreased with the deepening of the aging degree. Because in the short-term aging, asphalt aging to generate carbonyl and sulfoxide group. During UV aging, the carbonyl and sulfoxide groups decompose to form long chains or rings, and the aromatics and colloid converted to asphaltenes, promoting the further aging of asphalt [45]. Therefore, the preventing formation of C=O and S=O and the synthesis of long chain can delay the asphalt aging to contribute to the sustainable development of the asphalt pavement [46].

**Figure 10:**
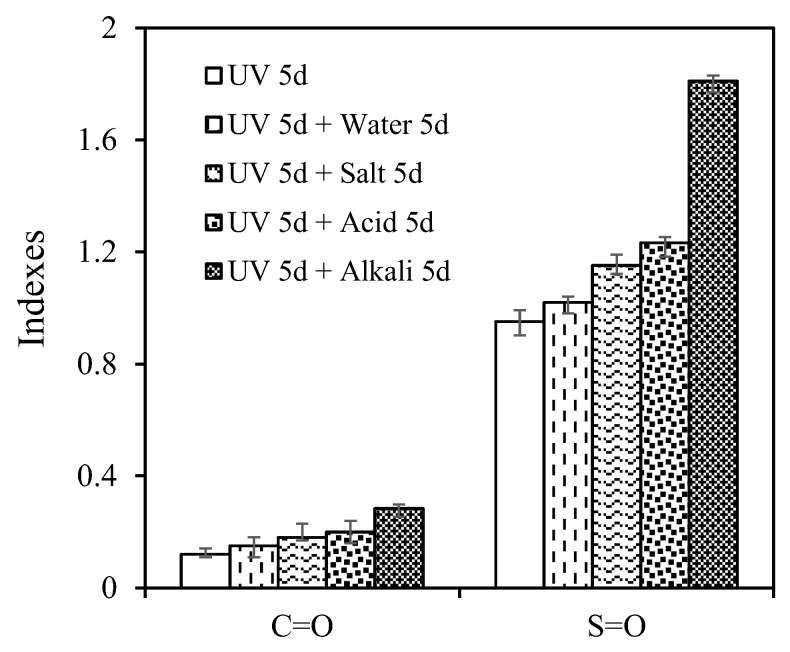
*I_C=O_* and *I_S=O_* of asphalt after the multiple aging of UV and aqueous solution.

**Figure 11:**
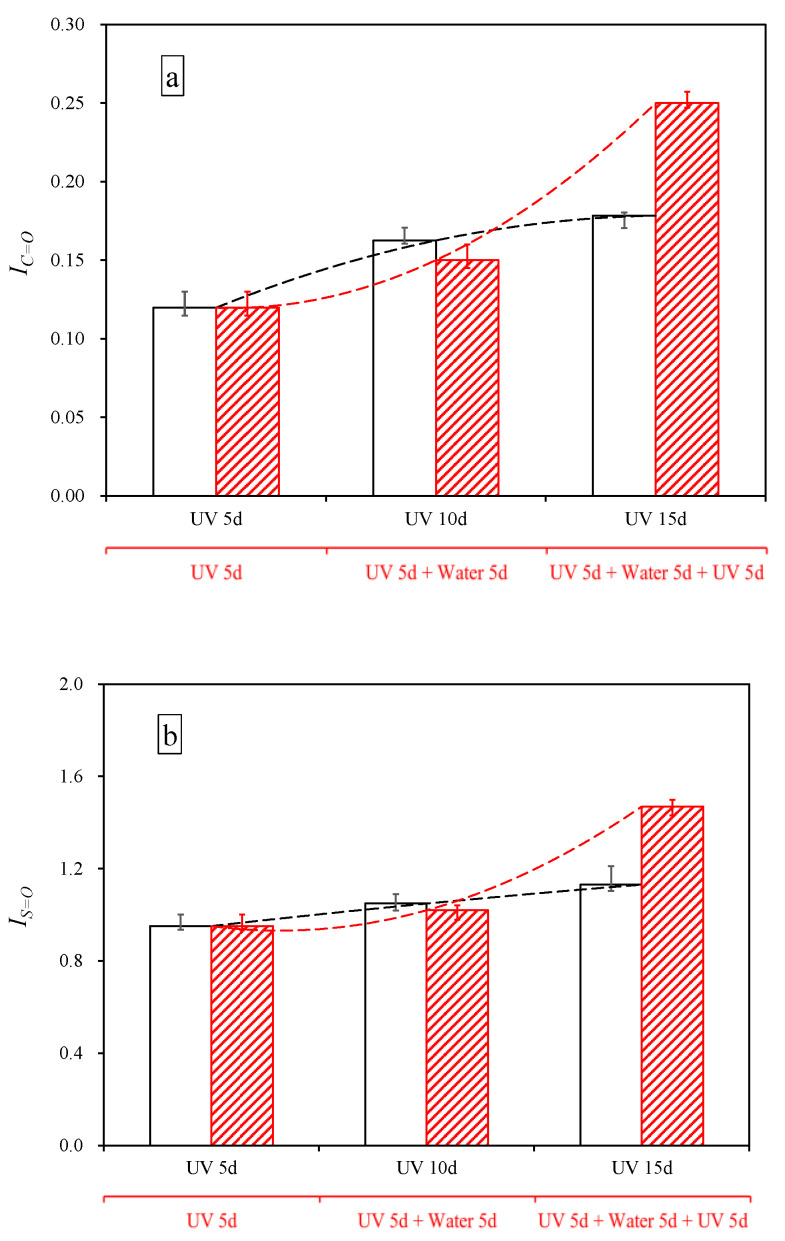
*I_C=O_* and *I_S=O_* of asphalt after the aging cycle of UV and water. (**a**) *I_C=O_*, (**b**) *I_S=O_*.

After the multiple aging of UV and aqueous solution, the *I_C=O_* and *I_S=O_* of asphalt are illustrated in Figure 10. After immersion in water, saline solution, acid solution and alkali solution, the *I_C=O_* increased by 25.31%, 50.38%, 67.08% and 136.42%, while the *I_S=O_* increased by 7.22%, 21.02%, 29.60% and 90.27%. This increment was greater than the increment when UV and aqueous solution are treated asphalt simultaneously, indicating that sequential treatment has a greater impact on asphalt than simultaneous treatment [18]. Aqueous solution could accelerate the asphalt oxidation after UV aging, and the order of the effect degree is as follows: alkali > acid > salt > water. Figure 11 shows the *I_C=O_* and *I_S=O_* of asphalt after aging cycle of UV and water. The sample (UV 5d + water 5d) had the lower *I_C=O_* and *I_S=O_* than the sample (UV 10d), denoting water had the less effect on chemical structure than UV. However, the *I_C=O_* and *I_S=O_* of sample (UV 5d + water 5d + UV 5d) were greater than that of sample (UV 15d). Moreover, from Table 7, it can be seen that the rangeability *I_C=O_* and rangeability *I_S=O_* between sample (UV 5d +water 5d + UV 5d) and sample (UV 5d + water 5d) was 2.5 and 4.5 times as much as that between the sample (UV 10d) and the sample (UV 5d), respectively. It shows that water could increase the sensitivity of asphalt chemical structure to UV. That agreed with the result of existing studies [47].

## 4. Conclusions

The high-temperature rutting resistance and low-temperature cracking resistance of asphalt under multiple conditions of heat, UV and aqueous solution were investigated by DSR test. The related mechanisms of changes in aging performance were discussed by the characterization of the chemical structure and element composition.

- Heat can increase the rutting factor, RAI and S, and decline the m-value to improve the high-temperature rutting resistance and weaken the low-temperature cracking resistance. According to the results of the EA test and FTIR test, the *f_A_*, *C_I_*, *I_C=O_* and *I_S=O_* increased after thermo-oxidative aging. It can be explained by the fact that thermo-oxidative aging causes the saturated hydrocarbon and the aromatic ring substance with the higher condensation degree increase to form more polar components. The addition of UV further deepens asphalt aging, and the sensitivity of physicochemical properties to UV decreased with the deepening of aging.
- Aqueous solution can further affect the rheological properties of samples aged by UV. Due to the dissolution and migration of polar components, the rutting resistance of asphalt samples was weakened but the cracking resistance was improved slightly after immersion in water, saline solution and acid solution, whereas the opposite pattern appears in samples suffered from alkali solution due to the saponification reaction. The order of influence of the degree of aqueous solution on UV-aged asphalt is as follows: alkali > acid > salt > water.
- Water has the smaller effect on element composition and chemical structure, but water can increase the sensitivity of physicochemical properties to UV.

This study explored the short-term cycling effect of heat, light and water on asphalt to provide an idea for simulation testing and anti-aging technology of asphalt under multiple aging conditions. The findings of this study also help to restore the lost properties of the binder from RAP, showing the significant economic and environmental outcomes. In future research, we will use the SARA test to investigate the influence of multiple aging conditions on the four components, and further explore the evolution of asphalt performance under multiple aging conditions.

## Figures and Tables

**Table 1:** Physical properties of 70 A.

Physical Properties	Units	70 A	Standards
Penetration (25 °C, 100 g, 5 s)	0.1 mm	72.4	ASTM D-5 [23]
Softening point	°C	49.6	ASTM D-36 [24]
Ductility (10 °C/5 °C)	cm	>100	ASTM D-113 [25]
Solubility (trichloroethylene)	%	99.5	ASTM D-2042 [26]

**Table 2:** Relevant information of instruments.

Test	Instruments	Origin	Test Parameters
DSR [27]	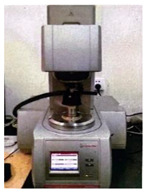	Smartpave 102	Stain: 0.5%
			Frequency: 10 rad/s
		Anton Paar Co., Ltd.	Temperature: 30–80 °C
			Heating rate: 2 °C/min
		Ostfildern, Germany	Plate diameter: 25 mm
			Plate gap: 1 mm
BBR [28]	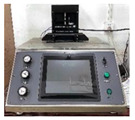	SYD-0627	Load: 980 ± 50 mN
		Shanghai Changji Geological Instrument Co., Ltd.	Temperature: −6, −12, −18 °C
		Shanghai, China	Span length: 102 mm
EA [29]	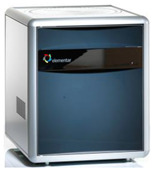	Vario EL Cube	Mode: C/H/N/S
		Elementar Analysensysteme GmbH	
		Langenselbold, Germany	
FTIR [30]	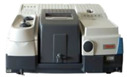	Nicolet 6700	Chip: KBr
		Thermo Fisher Scientifific	Scanning range: 4000–400 cm^−1^
		Waltham, MA, USA	Scan time: 64 times

**Table 3:** Element content of asphalt after the multiple aging of heat and UV (%).

Samples	C	H	N	S	O
Virgin	82.98	10.27	0.73	4.43	1.59
TFOT	82.94	10.24	0.72	4.38	1.72
UV 5d	82.86	10.21	0.72	4.37	1.84
UV 10d	82.86	10.20	0.72	4.36	1.86
UV 15d	82.67	10.17	0.72	4.34	2.10

**Table 4:** Rangeability of *C_I_* after the multiple aging.

Samples	Δ*C_I_*
Virgin/TFOT	0.0009
TFOT/UV 5d	0.0007
UV 5d/UV 10d	0.0004
UV 10d/UV 15d	0.0001
UV 5d/UV 5d + water 5d	0.0001
UV 5d + water 5d/UV 5d + water 5d + UV 5d	0.0005

**Table 5:** Element content of asphalt after the multiple aging of UV and aqueous solution (%).

Samples	C	H	N	S	O
UV 5d	82.86	10.21	0.72	4.37	1.84
UV 5d + water 5d	82.86	10.21	0.71	4.34	1.88
UV 5d + salt 5d	82.86	10.20	0.71	4.34	1.89
UV 5d + acid 5d	82.85	10.19	0.70	4.33	1.93
UV 5d + alkali 5d	82.84	10.18	0.69	4.32	1.97

**Table 6:** Element content of asphalt after the aging cycle of UV and water (%).

Samples	C	H	N	S	O
UV 5d	82.86	10.21	0.72	4.37	1.84
UV 5d + water 5d	82.86	10.21	0.71	4.34	1.88
UV 10d	82.86	10.20	0.72	4.36	1.86
UV 15d	82.67	10.17	0.72	4.34	2.10
UV 5d + water 5d + UV 5d	82.61	10.16	0.72	4.35	2.16

**Table 7:** Rangeability of *I_C=O_* and *I_S=O_* after the multiple aging.

Samples	Δ*I_C=O_*	Δ*I_S=O_*
Virgin/TFOT	0.06	0.21
TFOT/UV 5d	0.05	0.14
UV 5d/UV 10d	0.04	0.10
UV 10d/UV 15d	0.02	0.08
UV 5d/UV 5d + water 5d	0.03	0.07
UV 5d + water 5d/UV 5d + water 5d + UV 5d	0.10	0.45
